# Electronic and optical properties of perovskite compounds MA_1−*α*_FA_*α*_PbI_3−*β*_X_*β*_ (X = Cl, Br) explored for photovoltaic applications

**DOI:** 10.1039/c8ra08189a

**Published:** 2019-03-01

**Authors:** Junli Chang, Hong Chen, Guangzhao Wang, Biao Wang, Xiaorui Chen, Hongkuan Yuan

**Affiliations:** School of Physical Science and Technology, Southwest University Chongqing 400715 People’s Republic of China chenh@swu.edu.cn; Key Laboratory of Luminescent and Real-Time Analytical Chemistry, Ministry of Education, College of Chemistry and Chemical Engineering, Southwest University Chongqing 400715 People’s of Republic of China; School of Electronic Information Engineering, Yangtze Normal University Chongqing 408100 China

## Abstract

As outstanding light harvesters, solution-processable organic–inorganic hybrid perovskites (OIHPs) have been drawing considerable attention thanks to their higher power conversion efficiency (PCE) and cost-effective synthesis relative to other photovoltaic materials. Nevertheless, their further development is severely hindered by the drawbacks of poor stability and rapid degradation in particular. First-principles calculations based on density functional theory (DFT) are hence performed towards the perovskite compounds MA_1−*α*_FA_*α*_PbI_3−*β*_X_*β*_ (X = Cl, Br), with the aim of exploring more efficient and stable OIHPs. In addition to that, a hybrid density functional is adopted for exact electronic properties, and their band structures indicate that the doped series are all direct band-gap semiconductors. Moreover, the defect formation energies indicate that the stability of perovskite compounds can be significantly enhanced *via* ion doping. Meanwhile, it is unveiled that the optical performance of the doped perovskite series is also effectively improved through ion doping. Therefore, the investigated perovskite compounds MA_1−*α*_FA_*α*_PbI_3−*β*_X_*β*_ (X = Cl, Br) are promising candidates for enhancing solar-energy conversion efficiency. Our results pave a way in deeper understanding of the inherent characteristics of OIHPs, which is useful for designing new-type perovskite-based photovoltaic devices.

## Abstract 2

The absorption performance of perovskite CH_3_NH_3_PbI_3_ can be significantly improved *via* mono-, or co-doping of organic cations and halide ions.

## Graphical abstract

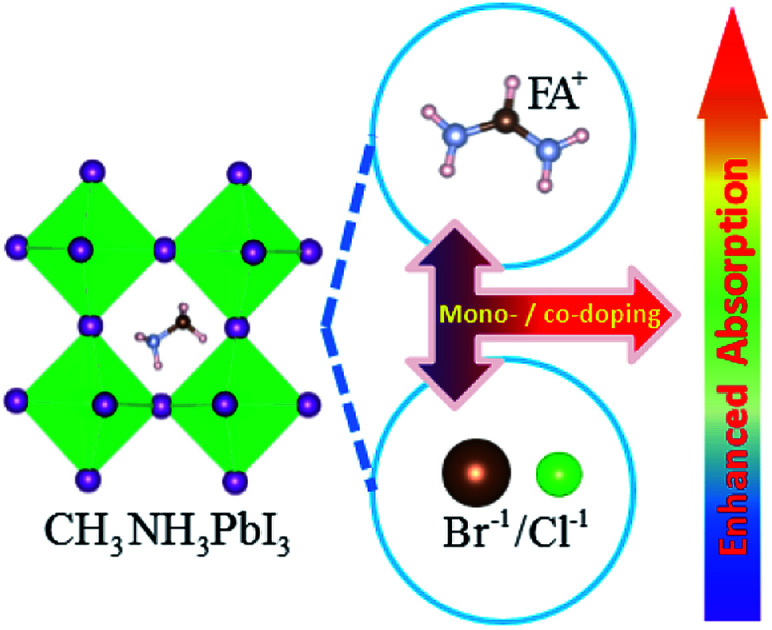

## 1 Introduction

Earth-abundant organic–inorganic hybrid perovskites (OIHPs), as new emerging photovoltaic materials, have attracted increasing interest in the last few years, due to their extraordinary performance in the development of cost-effective perovskite solar cells (PSCs). Their advantages include facile solution processability, benign grain boundaries and low trap-state density.^1–4^ It is especially worth emphasizing that carriers in OIHPs have superior performance including small effective mass, long lifetime and diffusion length in the micrometer level.^3,5–7^ Also, these hybrid materials can be readily fabricated using a variety of techniques such as spin coating,^8,9^ dip coating,^10,11^ vapour deposition,^12–14^ and thermal evaporation.^15^ In other words, these processing methods constitute so-called versatile photovoltaic technology. In accordance with the well-known cell efficiency chart provided by the American National Renewable Energy Laboratory (NREL), the PCE of PSCs has been rapidly exceeding 23% over the past years.^16^ Besides, perovskites can also be utilized in photodetectors, light-emitting diodes (LEDs) and so on.^17–19^ Owing to these predominant characteristics, perovskite-based semiconductors are expected to be the primary devices achieving solid state solar-energy conversion application in the near future. So deepening fundamental understanding towards the structural and electronic properties of OIHPs is of paramount importance for a wide range of photovoltaic applications.

Methylammonium lead halide perovskites were first introduced with the PCE of only 3.8% in 2009, as visible-light sensitizers in conjunction with liquid electrolyte.^20^ Although the resulting device had low PCE and poor stability, it was still considered as a milestone in the development of photovoltaic applications. From then on, the photoelectric conversion efficiency of perovskite-based solar cells was rapidly enhanced, and the stability was improved significantly. Quantum-dot-sensitized solar cells were successfully synthesized with the efficiency of 6.5%; nevertheless, the stability issue, arising from electrolyte dissolution, still existed.^21^ At that time, pure inorganic perovskite CsSnI_3_, as a hole conductor, was utilized to fabricate all-solid-state solar cells, achieving a sustainable conversion efficiency of 8.5%.^22^ Subsequently, perovskite solar cells with the higher PCE of 9.7% were soon realized. Interestingly, the first all-solid-state-solar-cells, by virtue of the hole-conductor spiro-MeOTAD, could sustainedly work for over 500 h under ambient air without encapsulation, which means that the stability of perovskite-based solar cells was preliminarily solved.^23^ Moreover, it was experimentally demonstrated that perovskites can also be used as an electron transport layer or hole transport layer in addition to being used for light harvesting.^24,25^ From then on, the research on OIHPs turned a new leaf. The efficiency of solar cells based on OIHPs was strikingly enhanced in the following years, achieving a record-breaking PCE of 23.7% to date.^16^ The velocity of ascension is unprecedented in the field of photovoltaic application. Significant efforts are being devoted to pursuing superior optical performance by means of ion doping or hetero-structured composites. It is worth emphasizing that a long diffusion length is crucial to improving cell performance, because of the resulting low carrier recombination. So far, it has been experimentally demonstrated that the diffusion length in the perovskite-based compound MAPbI_3−*x*_Cl_*x*_ can reach up to 1 micrometer, which is significantly longer than that in pure MAPbI_3_.^5^ Additionally, it has been verified that organic-cation doping in MAPbI_3_ can effectively reduce the band gap, though the band edges are not primarily populated by the electronic states of organic cations.^26–28^ Motivated by this progress, in this paper the electronic properties and optical capability of MA_1−*α*_FA_*α*_PbI_3−*β*_X_*β*_ (X = Cl, Br) are explored theoretically for optimal optical absorption. To our knowledge, this topic remains uncertain for the time being, so it is the main motivation for the present study.

**Fig. 1:**
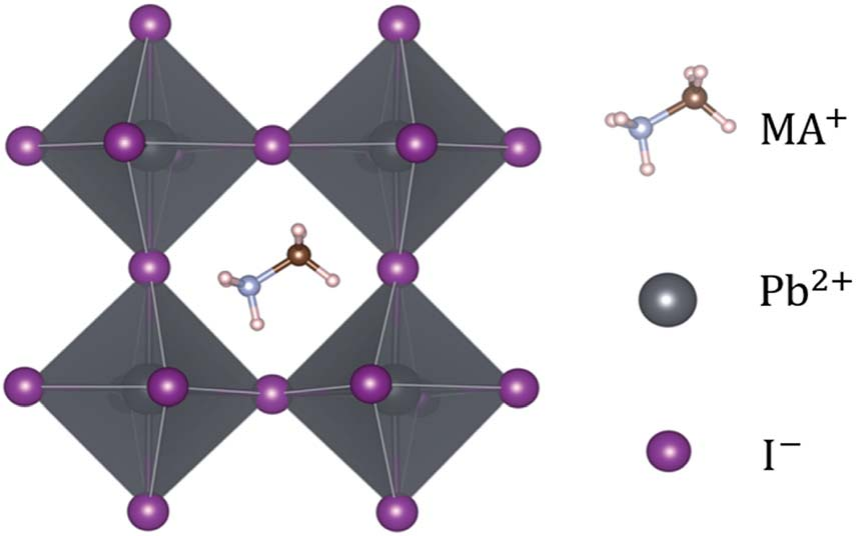
A typical perovskite, MAPbI_3_, along the [100] direction.

OIHPs, with the generic chemical formula of ABX_3_, are typically composed of a monovalent cation A (MA^+^, FA^+^ or Cs^+^, where MA = methylammonium, CH_3_NH_3_^+^; FA = formamidinium, NH_2_CH=NH_2_^+^), a divalent metal ion B (Pb^2+^, Sn^2+^ or Ge^2+^) and a halide ion X (I^−^, Br^−^, Cl^−^). To gain more insight into OIHPs, we chose methylammonium lead iodide, MAPbI_3_, as a typical example. For MAPbI_3_, shown in Fig. 1, the synthesis and characterization was originally reported by Weber in 1978.^29^ It was found that the Pb^2+^ metal ion was coordinated by six iodide ions (I^−^), forming typical [PbI_6_]^4−^ octahedrons, while the organic MA cation was surrounded by twelve iodide ions, *i.e.*, an organic molecule of MA located at the center of four neighboring [PbI_6_]^4−^ octahedrons. Moreover, the structure and symmetry of MAPbI_3_ are highly related to ambient temperature, which has been experimentally confirmed. Specifically, the ideal cubic perovskite structure (space group: *Pm*3̄*m*) exists when the ambient temperature is higher than 330.4 K. The tetragonal phase (space group: *I*4/*mcm*) is displayed when the temperature is between 161.4 K and 330.4 K. MAPbI_3_ adopts an orthorhombic phase at temperatures below 161.4 K. One point to emphasize herein is that the cubic phase may be converted to the complicated *δ* phase (space group: *P*6_3_*mc*), by cooling liquid interface.^30,31^ It is not difficult to get the fact that the increase of temperature means the enhancement of symmetry. Naturally, MAPbI_3_ under ambient temperature should be in the tetragonal phase. It is the reason that pure MAPbI_3_ in the tetragonal phase was chosen as the investigated system.

In the present work we focus on the following properties of perovskite series MA_1−*α*_FA_*α*_PbI_3−*β*_X_*β*_ (X = Cl, Br), including the geometry structures, electronic properties and optical absorption in particular. Based on DFT, the stability of the mono- and co-doped perovskites are deduced, involving chemical potentials of component elements from the viewpoint of growth conditions. In light of the defect formation energies, binding energies, DOS and charge distribution, we preliminarily deduce the most favorable case among the perovskite compounds. Moreover, the corresponding absorption spectra are simulated and detailed analyses are presented.

## 2 Computational details

First-principles investigation based on DFT^32^ has been carried out for the present study by using the Vienna *ab initio* simulation package (VASP).^33,34^ It is noteworthy that no symmetry constraint was contained in all the calculations. The density functional reported by Perdew, Burke and Ernzerhof (PBE) under the generalized gradient approximation (GGA) was chosen to deal with the exchange–correlation interaction in the lattice relaxation^35,36^ and total energy calculations, while the electron–ion interaction was treated by means of the projected augmented wave (PAW) method.^37^ In particular, the conjugate gradient algorithm was chosen to optimize lattice volume and atomic positions. The valence electron configurations for the constituent elements are shown as follows: C (2s^2^ 2p^2^), N (2s^2^ 2p^3^), H (1s^1^), Pb (5d^10^ 6s^2^ 6p^2^), I (5s^2^ 5p^5^), Br (4s^2^ 4p^5^) and Cl (3s^2^ 3p^5^), respectively. A Γ-centered 4 × 4 × 4 Monkhorst–Pack grid was adopted for sampling the Brillouin zones of the investigated pseudo-cubic systems.^38^ Meanwhile, the convergence criteria were set as follows: one was that the total energy change per atom was less than 1 × 10^−5^ eV, and the others were that the Hellmann–Feynman force per atom was on a threshold of 0.02 eV Å^−1^ and a cutoff energy of 500 eV was adapted for the plane wave basis set. Moreover, it is imperative to employ a vdW correction for this class of hybrid materials.^39^ For the present work, the empirical pairwise corrections were hence chosen to characterize dispersion weak interactions in PBE + D2.^40^ Note that the difference of total energy, resulting from the denser *k*-point or higher cutoff energy, was smaller than 0.01, so the selected relaxation standards are accurate enough for the investigated systems.

Besides, the spin-orbit coupling (SOC) interaction has been demonstrated to have a significant impact on OIHPs and on heavy metals in particular.^41,42^ Specifically, the SOC results in the decrease of band gaps, arising from the splitting of the conduction band minimum. As is well known, the calculations based on DFT often underestimate the band gap, which has been proved to be an accidental coincidence. Naturally, it is necessary to consider the SOC in electronic property studies for OIHPs. In addition, HSE06, a screened hybrid functional proposed by Heyd, Scuseria and Ernzerhof (HSE) was utilized to obtain an exact simulation.^43,44^ The electron–electron exchange energy consists of a long-range (LR) term and a short-range (SR) term, while the correlation energy refers to the PBE level. Based on the definition of HSE06, the exchange–correlation energy is given by

1 *E*^HSE^_XC_ = *ηE*^SR^_X_(*μ*) + (1 − *η*)*E*^PBE,SR^_X_(*μ*) + *E*^PBE,LR^_X_(*μ*) + *E*^PBE^_C_,

where *η* indicates the mixing coefficient and *μ* denotes the screening parameters. In the present work, both parameters were set to be 0.38 and 0.2 Å^−1^ to match the experimental values of lattice parameters and band gap for pure MAPbI_3_. Meanwhile, considering the limitation of computer resources, lower convergence criteria were applied, including a gamma-centered mesh of 3 × 3 × 3 for Brillouin zone sampling, a cutoff energy of 400 eV for plane wave basis and the total energy change per atom of 1 × 10^−4^ eV. The electronic properties of MA_1−*α*_FA_*α*_PbI_3−*β*_X_*β*_ (X = Cl, Br) involving DOS and PDOS were conducted in HSE + SOC. However, band structures and absorption spectra for doped compounds were simulated in PBE + D2 due to limited resources. With regard to pure MAPbI_3_, the simulated band gap in HSE + SOC reproduces accurately the reported observed experimental value. From this point of view, the results and discussion on the perovskite compounds MA_1−*α*_FA_*α*_PbI_3−*β*_X_*β*_ (X = Cl, Br) are reasonable and reliable in the paper.

## 3 Results and discussion

### 3.1 Geometric structure

To probe the intrinsic characteristics of the OIHP materials, we here adopt the tolerance factor, proposed originally by Goldschmidt,^45,46^ to analyze the geometry structures of perovskites. It is noteworthy that the organic cation is herein treated as a packed rigid sphere, then perovskite geometry is preliminarily determined by virtue of the tolerance factor. For a typical perovskite of ABX_3_, the definition of the tolerance factor is given in the following form,^45,46^2
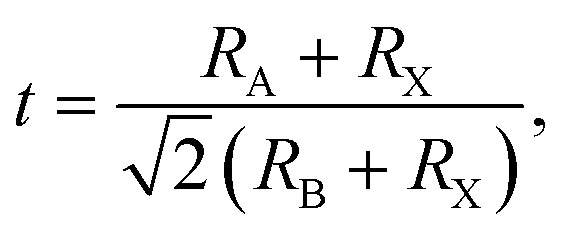
where *R*_A_, *R*_B_ and *R*_X_ indicate the ionic radii of the constituent elements A, B and X, respectively. It is hence noticeable that the tolerance factor is directly dependent on the radius ratios of organic cation and metal ion to halide ion. Concerning halide perovskites, the tolerance factor should be subject to the restrictive condition of 0.813 < *t* < 1.107,^47^ which implies that the ionic radius of the organic cation must be larger than that of the metal ion. Moreover, in light of empirical conditions, it is imperative that the range of the tolerance factor should be narrowed to 0.97 < *t* < 1.03 for ensuring the formation of the ideal cubic perovskites.^48^ In other words, when the tolerance factor is lower than 0.97, halide perovskites take on a trend to lower symmetry involving tetragonal, orthorhombic, trigonal and even hexagonal, which is arising out of cooperative octahedron distortion. Additionally, the octahedral factor is also an important index, with the following expression,^49^3
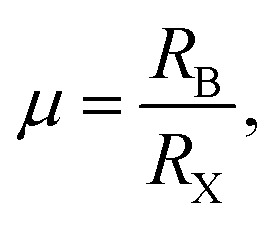
with the ionic radii *R*_B_ and *R*_X_. The octahedral factor is often in the range of 0.377 < *μ* < 0.895, however specifically for the formation of halide perovskites, the lowest limit of *μ* is actually 0.442; it has been experimentally demonstrated that an unstable perovskite can be synthesized with an octahedral factor lower than 0.442 even though the system has a reasonable tolerance factor.^49^ The tolerance factor and octahedral factor are hence rated as the necessary, but not sufficient, conditions for the formation of halide perovskites. In other words, crystallization stability and possible structures can be preliminarily deduced from the aforementioned theoretical definitions. It is noteworthy to emphasize that constituent ions are treated as packed rigid spheres with radii of *r*_MA_^+^ = 1.8 Å,^50^*r*_FA_^+^ = 1.9–2.2 Å,^51–53^, *r*_Pb_^2+^ = 1.19 Å, *r*_I_^−^ = 2.2 Å, *r*_Cl_^−^ = 1.81 Å and *r*_Br_^−^ = 1.96 Å.^47,54^ Obviously, it is not difficult to draw a conclusion that the tolerance factor becomes closer to 1 due to the injection of the large-size formamidinium cation or small-size Br/Cl ions, which suggest that the stability is effectively enhanced by the above ion doping.

**Fig. 2:**
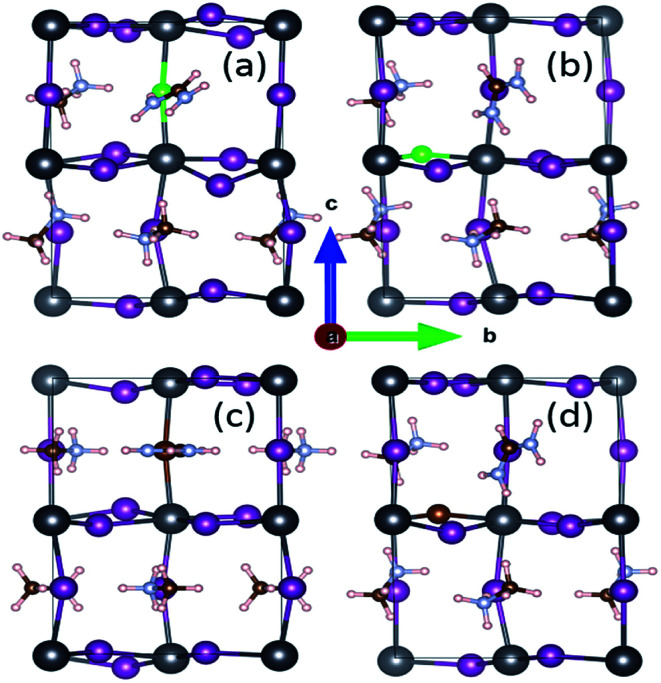
The investigated structures of perovskite compounds FACl-a (a), FACl-e (b), FABr-a (c) and FABr-e (d) in the [100] view. Detailed instructions on the cell geometry are presented in the text. The inorganic framework is composed of Pb (black), I (violet), Br (brown) and Cl (green). The others are organic cations (polar small molecule), including MA^+^ (CH_3_NH_3_^+^) and FA^+^ (NH_2_CH=NH_2_^+^).

For the archetype MAPbI_3_ with 48 atoms, the optimized lattice parameters of *a* = 8.50 Å, *b* = 8.76 Å and *c* = 12.75 Å in Table 1 are obviously in good agreement with the reported experimental values of *a* = 8.85 Å, *b* = 8.85 Å and *c* = 12.64 Å,^31^ the maximum change not exceeding 3.95%. In the present work, we focus on mono-doping and co-doping in terms of FA-, Cl- and Br-ions. Specifically, mono-doping consists of FA-mono, Cl-mono and Br-mono. As for co-doping, there are four cases including FACl-a, FACl-e, FABr-a and FABr-e. Note that the labels a/e indicate that the doping site of the halide ion is located in the apical/equatorial direction within [PbI_6_]^4−^ octahedrons, as shown in Fig. 2. In other words, the FACl co-doped cases, involving FACl-a and FACl-e, and the FABr co-doped cases, including FABr-a and FABr-e, are specifically discussed in the paper. Obviously, the doping concentration is only 10% for those, which is defined as the ratio of the number of doped atoms and that of the system. Note that the MA/FA is here considered as a large atom. In addition to that, only one situation is taken into account for the mono-doped series because the difference resulting from doping sites is neglectable through comparison. The calculated lattice parameters of doped perovskites MA_1−*α*_FA_*α*_PbI_3−*β*_X_*β*_ (X = Cl, Br) are listed in Table 1. The last column, expansion, in Table 1 indicates the cell volume change arising from ion doping, based on the pure MAPbI_3_. It is noteworthy that the volume of all the ion-doped series has significantly shrunk, with the exception of FA-mono doped MA_1−*α*_FA_*α*_PbI_3_, which is apparently related to the large size of the FA cation. Moreover, MA_1−*α*_FA_*α*_PbI_3−*β*_X_*β*_ (X = Cl, Br) presents the largest contraction among all the doped series. Actually, it is revealed that co-doped FACl-a perovskite has superior optical absorption compared to the other doped series, and detailed analyses are provided later in the section of optical properties.

**Table 1:** The calculated lattice parameters, volume and expansion for the studied perovskite compounds in the PBE + D2 level

System	*a* (Å)	*b* (Å)	*c* (Å)	*α* (°)	*β* (°)	*γ* (°)	Volume (Å^3^)	Expansion (%)
MAPbI_3_	8.50	8.76	12.75	90.76	90.03	89.98	949.64	0
FA-mono	8.69	8.65	12.74	89.79	89.03	90.38	957.57	0.84
Cl-mono	8.57	8.75	12.50	89.33	91.01	89.68	936.74	−1.36
Br-mono	8.52	8.76	12.61	90.50	90.56	89.80	940.49	−0.96
FACl-a	8.74	8.65	12.33	90.38	89.97	90.79	931.56	−1.90
FACl-e	8.68	8.52	12.68	91.55	88.33	92.17	936.41	−1.39
FABr-a	8.70	8.65	12.42	88.19	90.16	90.52	933.90	−1.66
FABr-e	8.64	8.62	12.72	91.00	88.74	91.04	947.25	−0.25

### 3.2 Defect formation energy

The ground-state total energy for pure MAPbI_3_ in the tetragonal phase can be obtained *via* self-consistent calculation after the geometry optimization. The cell volume in the equilibrium state can be theoretically derived by means of fitting the total energies and volumes of its derivatives, based on the equation of state. We next concentrate on discussion of the defect formation energy in the neutral state. The resulting defect formation is closely related to the synthesizing and annealing conditions; therefore, the defect formation energy is primarily determined by the chemical potentials of host atoms directly reflected by the ambient environment. With respect to the investigated perovskite MAPbI_3_, its formation enthalpy can be expressed as follows:^55^

**Fig. 3:**
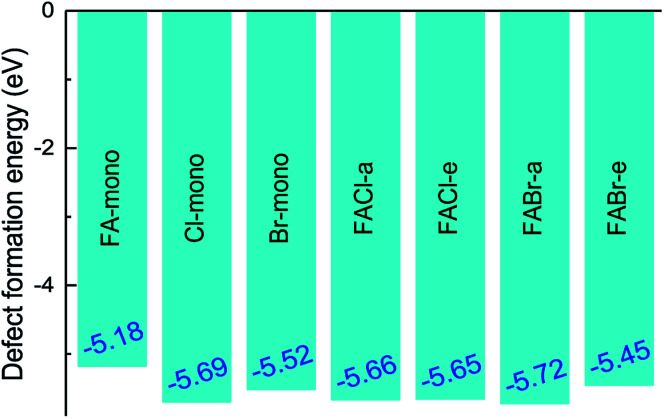
The defect formation energy for the doped perovskite-based compounds MA_1−*α*_FA_*α*_PbI_3−*β*_X_*β*_ (X = Cl, Br).

4 Δ*H*(MAPbI_3_) = *E*(MAPbI_3_) − *n*_*i*_*μ*_*i*_(bulk),

where the first term refers to the total energy of the prototype perovskite MAPbI_3_ with four MAPbI_3_ units. The parameter *n*_*i*_ denotes the number of component atoms in pure *E*(MAPbI_3_). Here, *μ*_*i*_(bulk) indicates the chemical potential of the component element in the equilibrium state, including MA(gas), Pb(bulk) and I(gas). Based on formation enthalpy, the defect formation energy for the doped perovskite series can be derived from the following form:

5 *E*_f_(def) = Δ*H*(doped) − Δ*H*(MAPbI_3_),

where Δ*H*(doped) and Δ*H*(MAPbI_3_), respectively, refer to the formation enthalpy of the doped series and pure MAPbI_3_ with the same number of atoms. Naturally, through combination of eqn (4) and (5), the defect formation energy can be further described as6
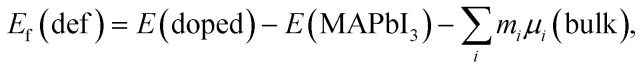
where *E*(doped) indicates the total energy for the doped compounds. The third item represents the energy arising from the difference in the constituent atom as compared to the prototype MAPbI_3_. The parameter *m*_*i*_ denotes the number of atoms injected or removed from a chemical reservoir. Specifically, it is a positive value for the added atoms and a negative value for the reduced atoms with the reference to the pure MAPbI_3_. As shown in Fig. 3, all the defect formation energies are negative, and among those the FA-mono has the maximal value of −5.175 eV, which implies that all the doped compounds can be experimentally synthesized. Moreover, it is noteworthy that the co-doped has lower formation energy than the mono-doped, which means that the former is more achievable to synthesize than the latter. Additionally, the binding energies of the FAX (X = Cl or Br) co-doped series can be used to deduce the relative stability compared to the mono-doped compounds, which can be simply calculated using the following equation,^55^

7 *E*_b_ = *E*_f_(MA_1−*α*_FA_*α*_PbI_3_) + *E*_f_(MAPbI_3−*β*_X_*β*_) − *E*_f_(MA_1−*α*_FA_*α*_PbI_3−*β*_X_*β*_).

**Fig. 4:**
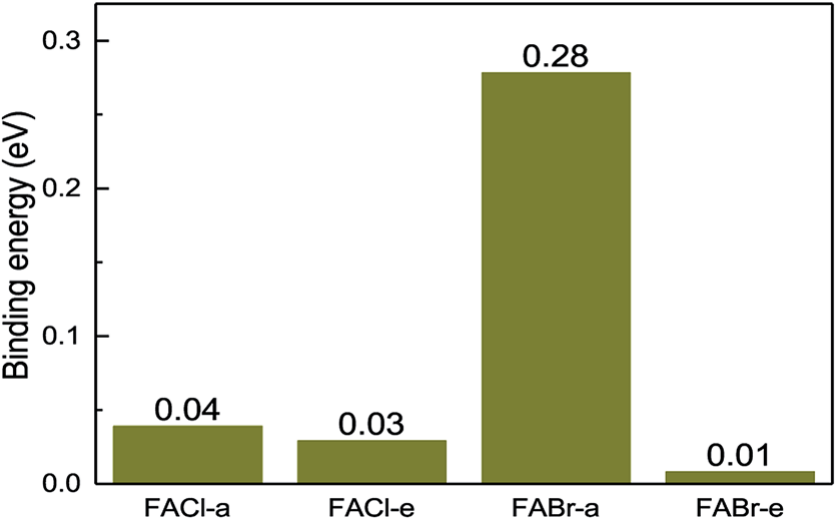
The binding energy for the co-doped series including FACl-a, FACl-e, FABr-a and FABr-e.

All the binding energies for the co-doped series are positive, which implies that the co-doped compounds are more stable than the mono-doped in Fig. 4. In addition to that, the FABr-a perovskite is the most stable among the co-doped series.

Furthermore, to ensure the synthesis of pure MAPbI_3_, the chemical potentials of component elements should satisfy the following equation:^56^

8 *μ*_MA_ + *μ*_Pb_ + 3*μ*_I_ = *μ*_MAPbI_3_(bulk)_,

where *μ*_MAPbI_3_(bulk)_ is equal to the total energy of pure MAPbI_3_. Meanwhile, the chemical potentials of component atoms in MAPbI_3_ are subject to the following expressions:

9a *μ*^min^_MA_ = *E*(MA_*n*_Pb_*n*_I_3*n*_) − *E*(MA_*n*−1_Pb_*n*_I_3*n*_),

9b *μ*^min^_Pb_ = *E*(MA_*n*_Pb_*n*_I_3*n*_) − *E*(MA_*n*_Pb_*n*−1_I_3*n*_),

9c *μ*^min^_I_ = *E*(MA_*n*_Pb_*n*_I_3*n*_) − *E*(MA_*n*_Pb_*n*_I_3*n*−1_).

Simultaneously, to avoid generating the impurity phases of MAI or PbI_2_, the following constraints must be satisfied,

10a *μ*_MA_ + *μ*_I_ < *μ*_MAI_,

10b *μ*_Pb_ + 2*μ*_I_ < *μ*_PbI_2__.

For simplicity, the changes of chemical potentials are defined as follows, with reference to the stable bulk phase in the equilibrium state:

11a Δ*μ*_MA_ = *μ*_MA_ − *μ*_MA(gas)_,

11b Δ*μ*_Pb_ = *μ*_Pb_ − *μ*_Pb(bulk)_,

11c
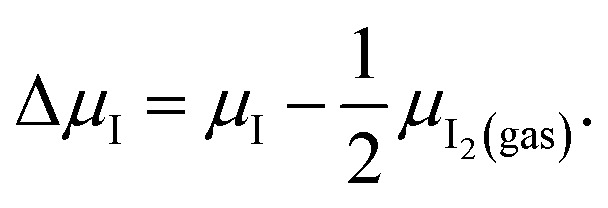


After that, the energies of the bulk phase are set to zero, and the ranges of Δ*μ* are further limited by:

12a Δ*μ*^min^_MA_ ≤ Δ*μ*_MA_ ≤ 0,

12b Δ*μ*^min^_Pb_ ≤ Δ*μ*_Pb_ ≤ 0,

12c Δ*μ*^min^_I_ ≤ Δ*μ*_I_ ≤ 0.

**Fig. 5:**
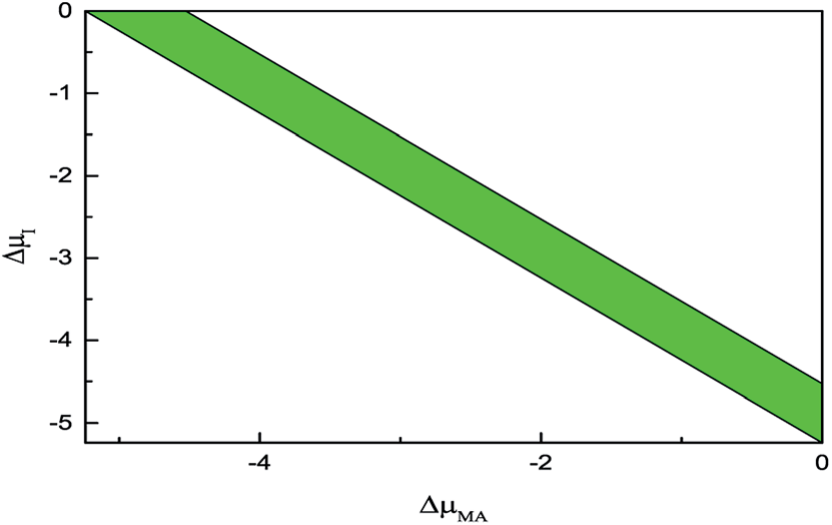
The physical accessible region of pure MAPbI_3_ in the coordinate plane defined by Δ*μ*_I_ and Δ*μ*_MA_.

Following the above constraints, Fig. 5 shows the physical accessible zone of the chemical potential of pure MAPbI_3_ in the coordinate plane defined by Δ*μ*_I_ and Δ*μ*_MA_. The narrow coloured region represents the low dissociation energy from MAPbI_3_ to MAI and PbI_2_. In other words, the growth conditions should be severely controlled to avoid the emergence of impurity phases of MAI or PbI_2_.

### 3.3 Electronic properties

#### 3.3.1 Density of states

**Fig. 6:**
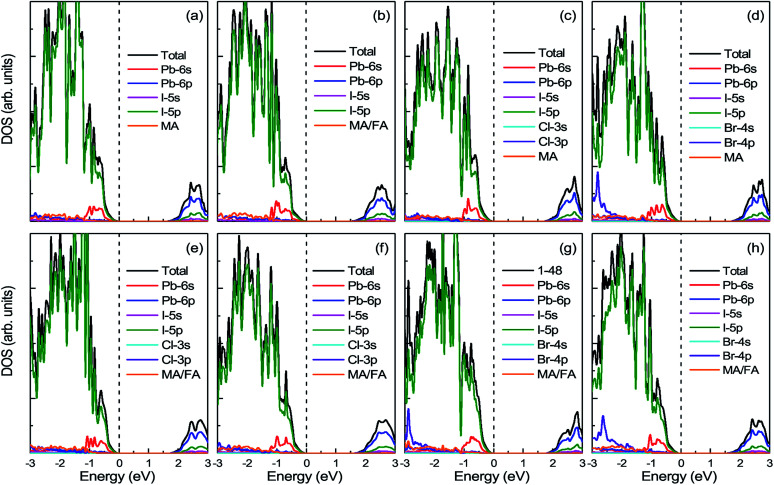
The calculated density of states (DOS) and projected density of states (PDOS) for prototype MAPbI_3_, mono-, and co-doped perovskite series MA_1−*α*_FA_*α*_PbI_3−*β*_X_*β*_ (X = Cl, Br). In the top panel, (a) represents the case of pure MAPbI_3_; (b)–(d), respectively, correspond to the cases of FA-mono, Cl-mono and Br-mono doped perovskite. In the bottom panel, (e) is for the FACl-a, (f) is for the FACl-e, (g) is for the FABr-a and (h) is for the FABr-e. The top of the valence band is chosen as the Fermi level and set to zero, as labeled in the black dashed lines.

Fig. 6 displays the DOS and PDOS of pure MAPbI_3_ and doped MA_1−*α*_FA_*α*_PbI_3−*β*_X_*β*_ (X = Cl, Br). It is noticeable that, for all the perovskites, the valence band maximum is primarily occupied by the I-5p orbital while the conduction band minimum is mainly dominated by the Pb-6p orbital, which is in excellent accordance with previous reports. Meanwhile, both the organic cations MA and FA have almost no contributions to the electronic orbitals with band edges. To explicitly compare the band-gap difference for the perovskite series, the VBM for all the perovskites is adopted as the Fermi level and set to energy zero. The pure MAPbI_3_ has a band gap of 1.64 eV, in good agreement with the experimental value, which suggests that the result in the paper is reliable. FABr-a has the widest band gap of 1.75 eV, while FA-mono has the narrowest band gap of 1.53 eV. In this respect, FA-mono should be a better choice to enhance optical performance, but this is not the case (related analyses are presented below). With respect to the stability of the perovskite series, the calculated defect formation energy is in accordance with the tolerance factor. Additionally, it is clearly revealed that the doped atoms have few contributions to the orbitals in the band edges. However, the band gaps of the perovskite compounds based on the pure MAPbI_3_ are significantly tuned *via* the lattice cell change, arising from ion doping. From this perspective, a conclusion can be drawn that FACl-a, as the top cell in a perovskite-based silicon tandem device, would be more favorable than the other doped compounds, due to a rational band gap of 1.70 eV.^57^

#### 3.3.2 Band structure

**Fig. 7:**
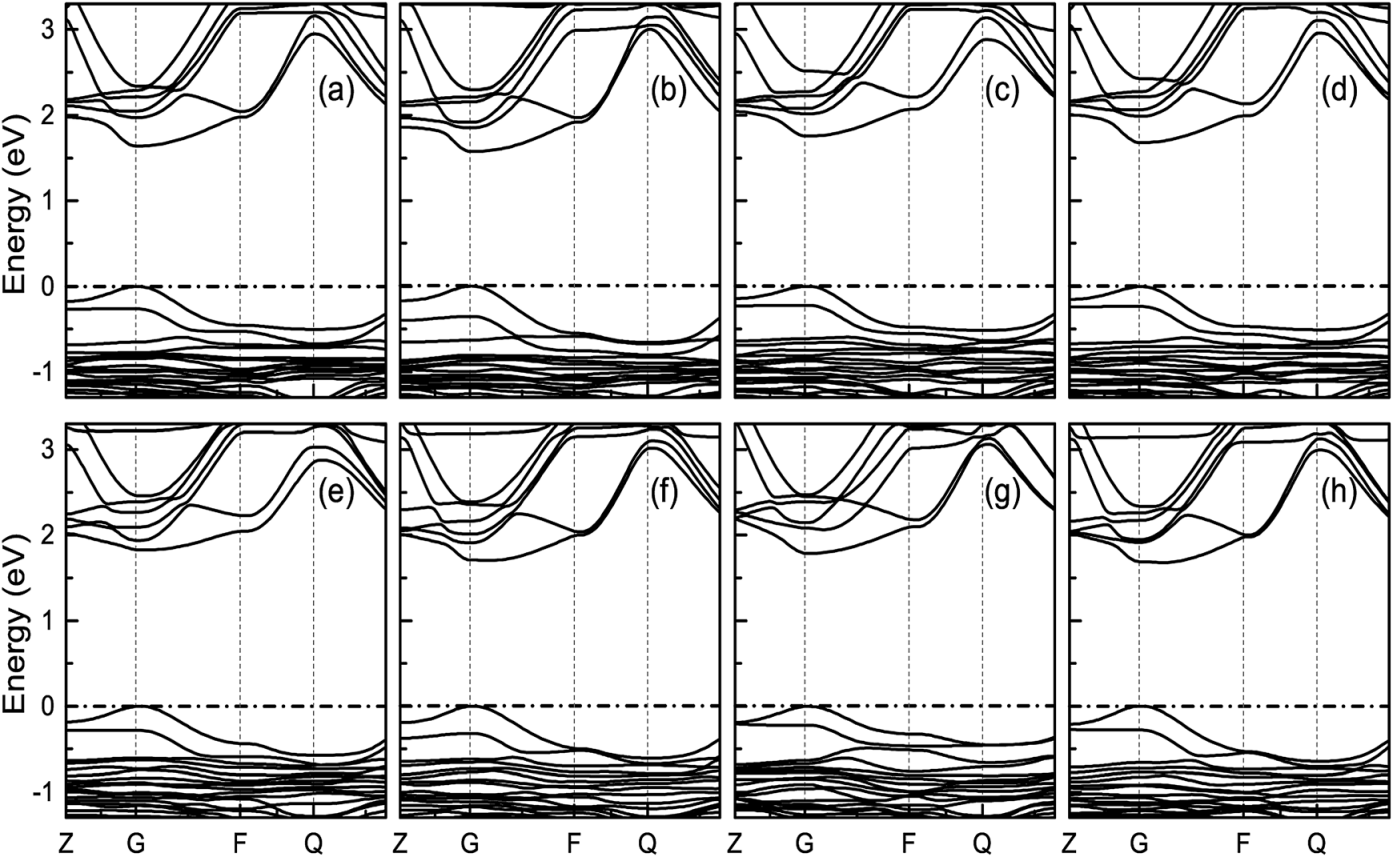
Band structure for the investigated perovskite series MA_1−*α*_FA_*α*_PbI_3−*β*_X_*β*_ (X = Cl, Br). The correspondence regular is the same as Fig. 6. The valence band maximum (VBM) is chosen as the Fermi energy of 0 eV and is presented in horizontal dashed-dot lines.

Fig. 7 indicates the band structures for the prototype MAPb_3_ and doped compounds MA_1−*α*_FA_*α*_PbI_3−*β*_X_*β*_ (X = Cl, Br). Specifically, the band structure for the prototype MAPbI_3_ is shown in Fig. 7(a). The high symmetry point coordinates are shown as *Z*(0.0, 0.0, 0.5), *G*(0.0, 0.0, 0.0), *F*(0.0, 0.5, 0.0) and *Q*(0.0, 0.5, 0.5), respectively. It is clearly unveiled that MAPbI_3_ is a direct band-gap semiconductor, which agrees well with previous studies.^54,58^ It is interesting that all the doping series are direct band-gap semiconductors. Therefore, it is deduced that the investigated compounds MA_1−*α*_FA_*α*_PbI_3−*β*_X_*β*_ (X = Cl, Br) are promising candidates, since it is more favorable for electron transitions in direct band-gap materials. Moreover, photo-generated carriers thermally relax to the VBM and CBM, hence, it is more favorable for transportation of those with small effective mass, in the following form,13
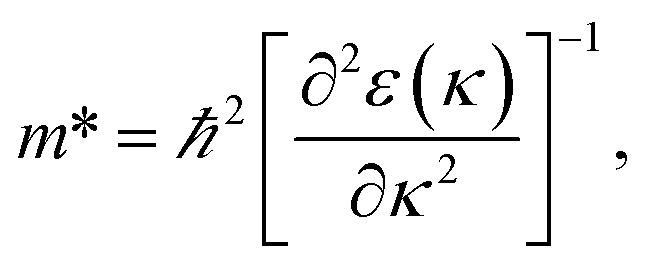
where the parameters are the reduced Planck constant *ℏ*, band edge eigenvalues *ε*(*κ*), and wave vector *κ*. To evaluate the reliability of the simulated effective mass, we introduce the reduced mass with the following definition:^54^14
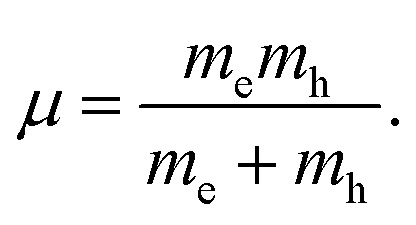


It is noteworthy that the obtained reduced mass for prototype MAPbI_3_ is comparable with that reported in ref. 59 and 60, which suggests that the simulation is reasonable. Table 2 lists the effective mass (along the *G*–*F* direction) and reduced mass for the doped perovskite compounds MA_1−*α*_FA_*α*_PbI_3−*β*_X_*β*_ (X = Cl, Br), in the unit of electron static mass (*m*_0_). It is clearly demonstrated that FA-mono and FABr-e are superior compared with the other doped systems.

**Table 2:** The calculated effective mass in the *G*–*F* direction and reduced mass for the investigated series

System	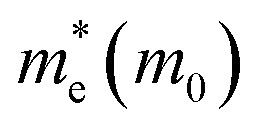	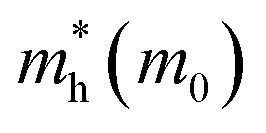	*μ*(*M*_0_)
Pure	0.16	0.47	0.12
FA-mono	0.14	0.40	0.10
Cl-mono	0.21	0.63	0.16
Br-mono	0.18	0.56	0.14
FACl-a	0.29	0.50	0.18
FACl-e	0.40	0.37	0.19
FABr-a	0.20	0.51	0.14
FABr-e	0.15	0.39	0.11

#### 3.3.3 Charge density distribution

**Fig. 8:**
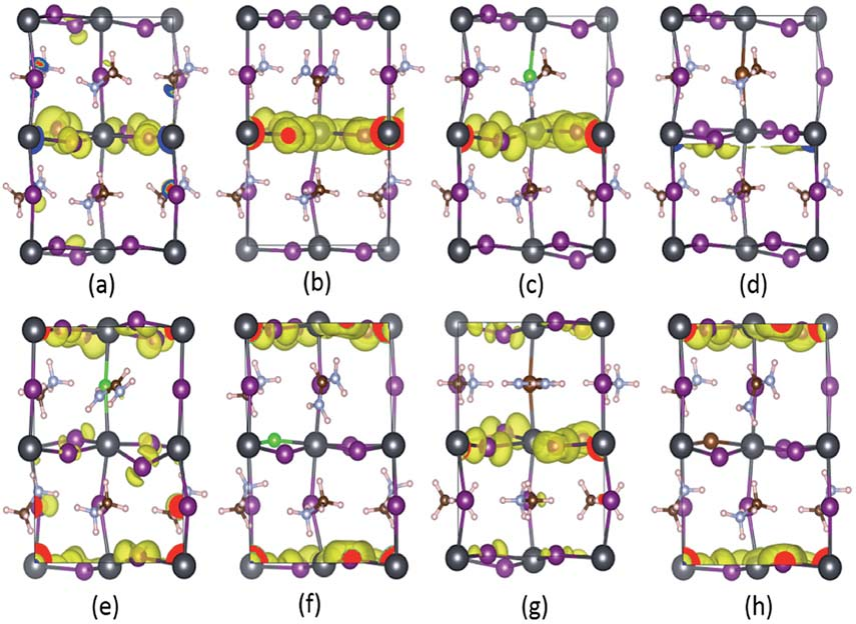
The simulated partial charge distribution of the VBM of the doped perovskite series with an iso-value of 0.00229 eÅ^−3^. Parts (a)–(h) correspond to pure, FA-mono, Cl-mono, Br-mono, FACl-a, FACl-e, FABr-a and FABr-e, respectively. The charge depletion area is in blue, while that of charge accumulation is in yellow. Note that the coordinate system herein is the same as that in Fig. 2.

To gain more intrinsic characteristics of the perovskite series, we examine the charge distribution of the VBM. As shown in Fig. 8, the VBM is dominated by the I-5p electronic orbital, which agrees well with the DOS analysis in Fig. 6. The charge distributions of the pure and mono-doped series are mainly localized inside the bulk, while those of the co-doped ones are localized at the outermost region, with the exception of the FABr-a. Obviously, it is more favorable for a photo-generated carrier (hole) to be transferred to the adjacent hole transport layer (HTL) in the latter case.^61^ In other words, the latter case is better for enhancing the separation efficiency of photo-generated carriers, with lower recombination. From this perspective, the energy loss resulting from charge transfer is smaller compared to the former case, which would directly help to enhance the PCE of solar cells.

### 3.4 Optical properties

To probe optical absorption properties for the doped MA_1−*α*_FA_*α*_PbI_3−*β*_X_*β*_ (X = Cl, Br), it is imperative to introduce the complex dielectric function. As is well known, the complex dielectric function depends on frequency and its imaginary part has the following form,^62^15
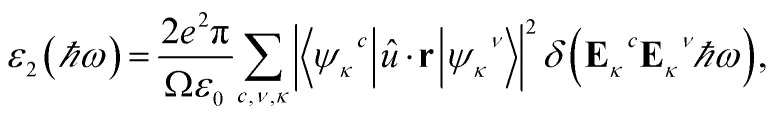
where the parameters are listed as follows. The wave functions of *Ψ_κ_^c^* and *Ψ_κ_^v^* denote the unoccupied state and occupied state at the *κ*-point reciprocal space, respectively. *û* indicates the polarization vector for the incident electronic field. The other symbols *ω*, *Ω* and *r* refer to the photon frequency, cell volume and momentum operator, respectively. The real part in the complex dielectric function can be readily obtained, according to the famous Kramers–Kronig relationship:^63^16
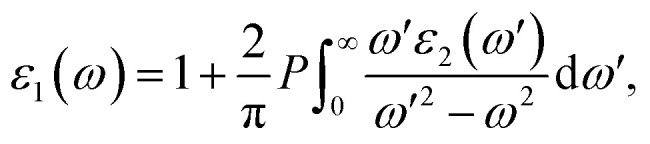
where *P* refers to the principal value of the integral. Consequently, the frequency-dependent optical absorption coefficient can be derived from the following equation,^64^17
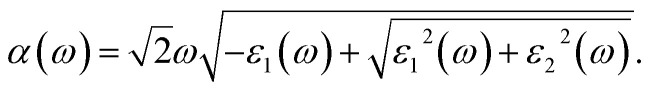


**Fig. 9:**
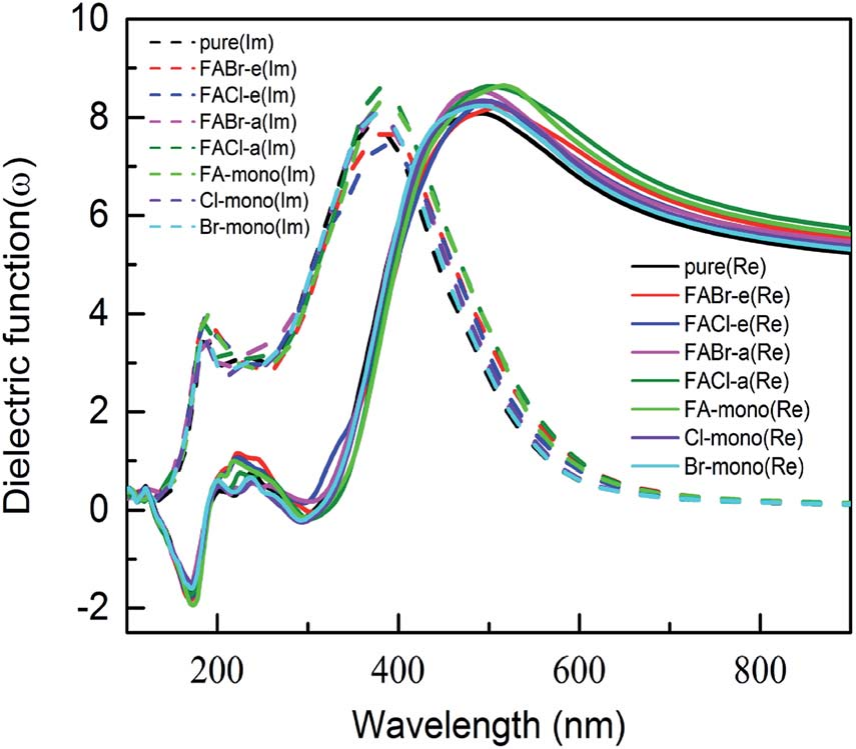
The calculated dielectric spectra for the prototype MAPbI_3_ and compounds MA_1−*α*_FA_*α*_PbI_3−*β*_X_*β*_ (X = Cl, Br). Note that the imaginary parts of the dielectric function are shown in dashed lines, and the real parts are presented in solid lines.

**Fig. 10:**
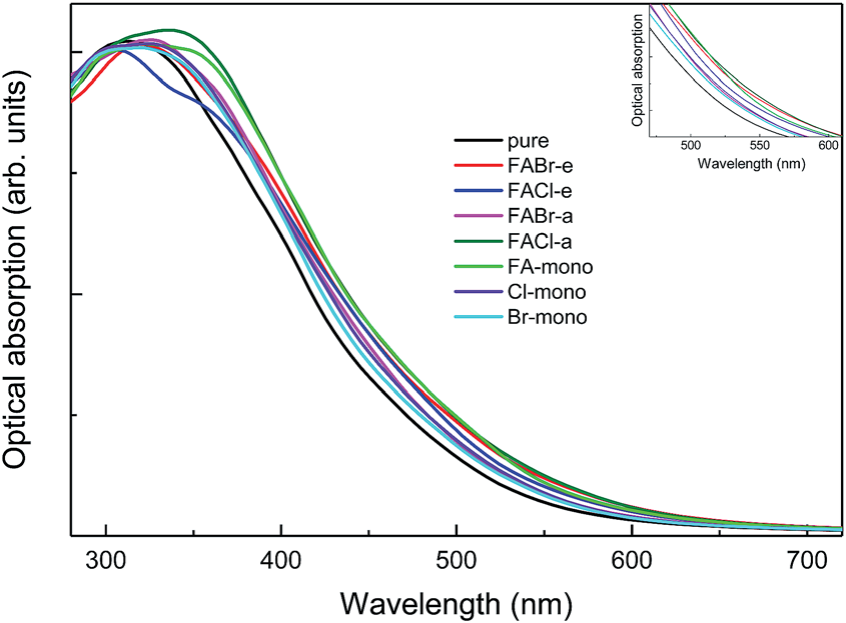
The absorption spectra for pure MAPbI_3_ and perovskite compounds MA_1−*α*_FA_*α*_PbI_3−*β*_X_*β*_ (X = Cl, Br). The black refers to the pure MAPbI_3_ and the other colors correspond to the doped series, as labeled in the legend. The inset shows the same absorption spectra in the specific range of 470–610 nm.

To probe optical harvesting capability, we next examined dielectric spectra for the investigated series MA_1−*α*_FA_*α*_PbI_3−*β*_X_*β*_ (X = Cl, Br). The imaginary parts and the real parts are respectively indicated in Fig. 9. As reported in ref. 65, with respect to the imaginary parts, it is apparent that two peaks exist at about 200 nm and 400 nm, and after that it presents a downward trend. In particular, for primitive MAPbI_3_, the simulated dielectric spectrum is well consistent with the previous report.^54^ Moreover, among the mono- and co-doping series, FACl-a has the maximum value, which is consistent with its superior optical absorption as shown in Fig. 10. As for the real parts shown in Fig. 9, two negative values appear at about 180 nm and 300 nm, which implies that the investigated compounds have strong absorption ability in the range of the ultraviolet spectrum. Additionally, all the doped perovskite compounds exhibit a better overlap with the archetype MAPbI_3_, in terms of imaginary parts and real parts. Consequently, it is implied that the perovskite compounds MA_1−*α*_FA_*α*_PbI_3−*β*_X_*β*_ (X = Cl, Br) have better solar energy harvesting ability in the range of the visible-light spectrum.

As shown in Fig. 10, it is interesting to find that the absorption spectra suggest that for both the mono- and co-doped compounds, the optical performance is significantly improved, over almost the whole visible-light range of 400–700 nm. The absorption spectra are enlarged in the inset of Fig. 10, and it is revealed that FA-mono and FACl-a are both superior compared to the other doped systems. Particularly, both of them have almost the same absorption coefficient as one another in the visible light range of 400–510 nm, while FACl-a displays higher optical absorption in the range of 510–700 nm. Meanwhile, charge distributions for all the doped series are clearly presented in Fig. 8. Consequently, the compound MA_1−*α*_FA_*α*_PbI_3−*β*_Cl_*β*_ (FACl-a) is rated as a better optical absorption material for photovoltaic applications.

## 4 Conclusions

In summary, a first-principles study, involving a hybrid density functional (HSE06), has been conducted to explore the electronic properties and optical performance of the perovskite compounds MA_1−*α*_FA_*α*_PbI_3−*β*_X_*β*_ (X = Cl, Br), based on pure MAPbI_3_ in the tetragonal phase. The calculations of defect formation energy indicate that it is advantageous to synthesize the doped series, with the exception of the FA-mono. For the co-doped series, four possible cases have been discussed in detail, and the calculations of binding energy imply that the stability of the co-doped series is significantly superior to that of the mono-doped ones. The physically accessible region has been clearly determined by the chemical potentials change of the organic cation MA and halide ion I. Additionally, the VBM primarily consists of the I-5p orbital while the CBM is mainly composed of the Pb-6p electronic state. It has further been confirmed that the band gaps of perovskite series MA_1−*α*_FA_*α*_PbI_3−*β*_X_*β*_ (X = Cl, Br) can be effectively tuned by means of ion doping, although organic cations are almost irrelevant to the electronic states in the band edges. Significantly, FA-mono has an ideal band gap among the doped compounds. However, its charge distribution in the VBM implies that it is unfavorable for the separation of photo-generated carriers. The FA-mono compound is hence excluded for constructing photovoltaic devices. Last but not least, absorption spectra suggest that the FACl-a is superior compared with the other doped systems, almost over in the range of the whole visible-light spectrum. This work has studied the inherent mechanism for MA_1−*α*_FA_*α*_PbI_3−*β*_X_*β*_ (X = Cl, Br) and, more importantly, also provides new insight for the development of new-category functional materials for photovoltaic conversion.

## Conflicts of interest

There are no conflicts to declare.

## Supplementary Material
